# Memantine Administration Enhances Glutamatergic and GABAergic Pathways in the Human Hippocampus of Alzheimer's Disease Patients

**DOI:** 10.1002/pmic.70006

**Published:** 2025-07-09

**Authors:** Ivo Fabrik, Rudolf Kupcik, Daniela Fabrikova, Marketa Chvojkova, Kristina Holubova, Kristina Hakenova, Martin Horak, Jiri Soukup, Monika Manethova, Robert Rusina, Radoslav Matej, Ales Ryska, Ondrej Soukup

**Affiliations:** ^1^ Biomedical Research Centre University Hospital Hradec Kralove Hradec Kralove Czech Republic; ^2^ National Institute of Mental Health Klecany Czech Republic; ^3^ Third Faculty of Medicine Charles University Ruska 87 Prague Czech Republic; ^4^ Institute of Experimental Medicine of the Czech Academy of Sciences Prague Czech Republic; ^5^ The Fingerland Department of Pathology University Hospital Hradec Kralove Hradec Kralove Czech Republic; ^6^ Department of Pathology Military University Hospital Prague Prague Czech Republic; ^7^ Department of Pathology First Faculty of Medicine, Charles University and General University Hospital in Prague Prague Czech Republic; ^8^ Clinic of Neurology Third Faculty of Medicine and Thomayer Hospital Prague Czech Republic; ^9^ Department of Pathology and Molecular Medicine Third Faculty of Medicine Charles University and Thomayer University Hospital Prague Czech Republic

**Keywords:** Alzheimer's disease, FFPE, glutamate receptors, hippocampus, memantine, metabolism, proteomics

## Abstract

One of the traditional treatments in Alzheimer's disease (AD) is administration of memantine, the NMDA receptor antagonist. However, the molecular mechanism of the complex memantine action and the impact on the hippocampal proteome in humans is unknown. In this study, hippocampal proteins extracted from formalin‐fixed paraffin‐embedded post mortem tissues obtained from healthy donors (*n* = 15), AD patients not treated with memantine (*n* = 11), and AD patients treated with memantine (*n* = 8) were investigated using tandem mass tag (TMT)‐based quantitative proteomics. Memantine medication induced subtle but distinct changes in the hippocampal proteome in AD patients. Although it did not prevent the metabolic and physiologic decline associated with AD pathology, memantine administration upregulated several mitochondrially encoded proteins and mitigated the proteomic pattern of activated phagocytes. Furthermore, memantine specifically enhanced the expression of postsynaptic glutamatergic and GABAergic receptors and components of the respective pathways without affecting presynaptic proteome. This suggests that memantine treatment in AD patients not only alleviates excitotoxic stress by inhibiting NMDA receptor activity, but also triggers broader adaptations in the synaptic signaling and plasticity.

**Abbreviations**
AD: Alzheimer's disease
AD + M: Alzheimer's disease + memantine
Aβ: amyloid beta
BCA: bicinchoninic acid assay
BM: basement membrane
FFPE: formalin‐fixed paraffin embedded
GABA: γ‐aminobutyric acid
GPCRs: G‐protein‐coupled receptors
H&E: hematoxylin & eosin
iGluRs: ionotropic glutamate receptors
LPS: lipopolysaccharide
mGluRs: metabotropic glutamate receptors
MHC: major histocompatibility complex
NMDARs: *N*‐methyl‐D‐aspartate receptors
RC: respiratory complex
TCA: tricarboxylic acid cycle
TEAB: triethylammonium bicarbonate buffer
TMT: tandem mass tag

1

Alzheimer's disease (AD) represents a serious health, social, and economic problem manifesting as a cognitive disorder affecting principally memory and executive functions. Although the underlying pathophysiological mechanisms resulting in clinical symptoms have been well described, the etiology of this disease has not yet been fully defined. Notable elements of the pathophysiology include hypofunction of the cholinergic system, glutamate‐induced excitotoxicity mediated by *N*‐methyl‐D‐aspartate receptors (NMDARs), inflammation and oxidative stress in the brain, accumulation of amyloid beta (Aβ) plaques extracellularly, and neurofibrillary tangles of phosphorylated tau protein intracellularly. Long‐term excitotoxicity associated with Aβ, together with tau and inflammation, is responsible for impairment of cellular metabolism leading to neuronal death and overall neurodegeneration [1].

Besides the novel monoclonal anti‐Aβ antibodies lecanemab and aducanumab [2], available treatments for cognitive decline are traditionally based on the administration of cholinesterase inhibitors and/or the NMDAR antagonist memantine [3]. Memantine, which prevents Ca^2+^ mediated excitotoxicity, is an uncompetitive antagonist with moderate potency and rapid strongly voltage‐dependent blocking kinetics [4]. Clinically, it is used against moderate and severe AD. In the United States, it is also widely used off‐label for mild AD; however, a small clinical benefit can be proved only for moderate‐to‐severe AD [5]. Memantine was reported to exert many pharmacological effects modulating, foremostly in pathological conditions, Aβ production and related toxicity, tau phosphorylation, insulin signaling, synaptic plasticity, long‐term potentiation, memory and cognition, autophagy, apoptosis, and neuronal proliferation [6]

There are two groups of glutamate receptors: ionotropic (iGluRs) and metabotropic glutamate receptors (mGluRs). The iGluRs mediate fast excitatory synaptic transmission and excitotoxicity in the CNS and are localized post‐synaptically, pre‐synaptically, or extra‐synaptically on neuronal and non‐neuronal cells including glial cells [7, 8]. The mGluRs are G‐protein‐coupled receptors (GPCRs) localized both pre‐synaptically and post‐synaptically, regulating synaptic plasticity and neuronal excitability [9]. In mammals, the iGluRs (NMDARs, AMPA receptors, kainate receptors, and δ‐receptors) are the main excitatory receptors in the CNS. Whereas AMPA and kainate receptors are extremely fast receptors activated by glutamate, the NMDAR is activated by glutamate and glycine or D‐serine as a co‐agonist. Pharmacologically, memantine is an NMDAR blocker with no or negligible efficacy on kainate, AMPA or glycine receptors [4], and with clinically irrelevant antagonistic activity on some nicotinic (e.g., α7) and 5‐HT_3_ receptors, irrelevant agonistic activity on D_2_ receptors in the striatum, and irrelevant action on voltage‐gated Ca^2+^ and K^+^ channels [6].

Thus, memantine administration blocks NMDA‐mediated signal transduction, and this inhibition consequently leads to complex cellular alterations manifesting in different protein expression and overall changes in the cellular metabolism and tissue function. Although there are many studies describing the proteomic changes associated with AD, such studies involving memantine are sparse, especially in humans. Indeed, the human meta‐analysis highlights AD‐associated changes in synaptic signaling and apoptotic and proteasomal catabolic processes, while confirming general dysregulation in oxidative phosphorylation, immune response, and extracellular matrix [10]. However, a study on the effects of memantine on a proteome using 3xTg‐AD mice chronically receiving memantine reported changes in cytoskeleton pathways and ErbB signaling in the hippocampus [11]. Furthermore, memantine also modulated more complex systems, for example, electron transport, cytoskeleton, ribosomes or calcium, and MAPK signaling in the cortex [12].

Thus, the aim of this study was to investigate how the complex action of memantine is reflected in protein expression in the human hippocampus. Although the effect on the expression of glutamatergic proteins is expected, the complex impact has never been studied using human tissues.

Those parts of the study involving humans have been approved by the Ethical Committee of the University Hospital Hradec Kralove (ref. 201906S15P). Animal experiments were approved by the Animal Care and Use Committee of the National Institute of Mental Health (MZDR 19618/2021‐5/OVZ). Human proteome was extracted from formalin‐fixed paraffin‐embedded (FFPE) *post mortem* hippocampal tissues from healthy donors (15, average age 64.9 ± 6.3 years), AD patients not treated with memantine (11, average age 81.8 ± 11.3), and AD patients treated with memantine (8, average age 78.5 ± 5.2) (Table S1) and analyzed by tandem mass tag (TMT)‐based quantitative proteomics (Figure 1A;). Histological samples of brain tissue were collected from the autopsy cases. The autopsies were performed in the period between 8 and 72 h after the death of the patient. All the samples were fixed in 4% buffered formaldehyde solution and processed in ethanol and xylene using an automated tissue processor according to the routine histopathological protocol for tissue samples. Finally, the sample was paraffin‐embedded and cut into 3 µm thick tissue sections for H&E and additional special staining. For the purpose of the study, whole FFPE tissue blocks or sections (samples AD+M 3–8) were deparaffinized and dried (Table S1). Proteins were extracted with Protein Extraction Buffer (Abcam) by homogenizing the tissues in a disperser, followed by heating at 105°C for 30 min and centrifugation. The protein concentration in the supernatants was determined by bicinchoninic acid (BCA) assay, and the respective protein amounts from each sample were combined with 2 × S‐trap lysis buffer (final concentration 5% sodium dodecyl sulfate/50 mM triethylammonium bicarbonate buffer [TEAB]). Samples were then alkylated with iodoacetamide, acidified with phosphoric acid, and mixed with 90% methanol/100 mM TEAB before digestion on S‐trap (Protifi) [13, 14] by Lys‐C/trypsin mixture (Promega). Peptides eluted from S‐trap were lyophilized, dissolved in 100 mM TEAB, and quantified by fluorometric peptide assay (Thermo). Samples were then labeled with the respective TMT10plex tags (Thermo), distributed into four multiplexes (each containing pooled reference sample, see Table S2), and desalted. Each TMT multiplex was fractionated by high pH reversed‐phase HPLC and individual fractions were analyzed by data‐dependent acquisition using UltiMate 3000 RSLCnano connected via Nanospray Flex ion source to Orbitrap Exploris 480 equipped with FAIMS Pro Duo interface (Thermo). Collected data were searched by MSFragger [15] against the Uniprot reference proteome for *Homo sapiens*, and spectral matches were rescored and validated by MSBooster and Percolator [16, 17]. ProteinProphet and Philosopher were used for protein inference and filtering of proteins at 1% FDR. TMT quantification was done by TMT‐Integrator using razor and unique peptides, and data were normalized across multiplexes using pooled reference sample [18]. The mass spectrometry proteomics data have been deposited to the ProteomeXchange Consortium (http://proteomecentral.proteomexchange.org) via the PRIDE partner [19] repository with the dataset identifier PXD052985. Memantine‐dependent expression of selected hippocampal proteins was validated by Western blot in a 5 × FAD transgenic mouse model of AD chronically receiving memantine. Further details regarding methods can be found in the Supporting Information.

**FIGURE 1:**
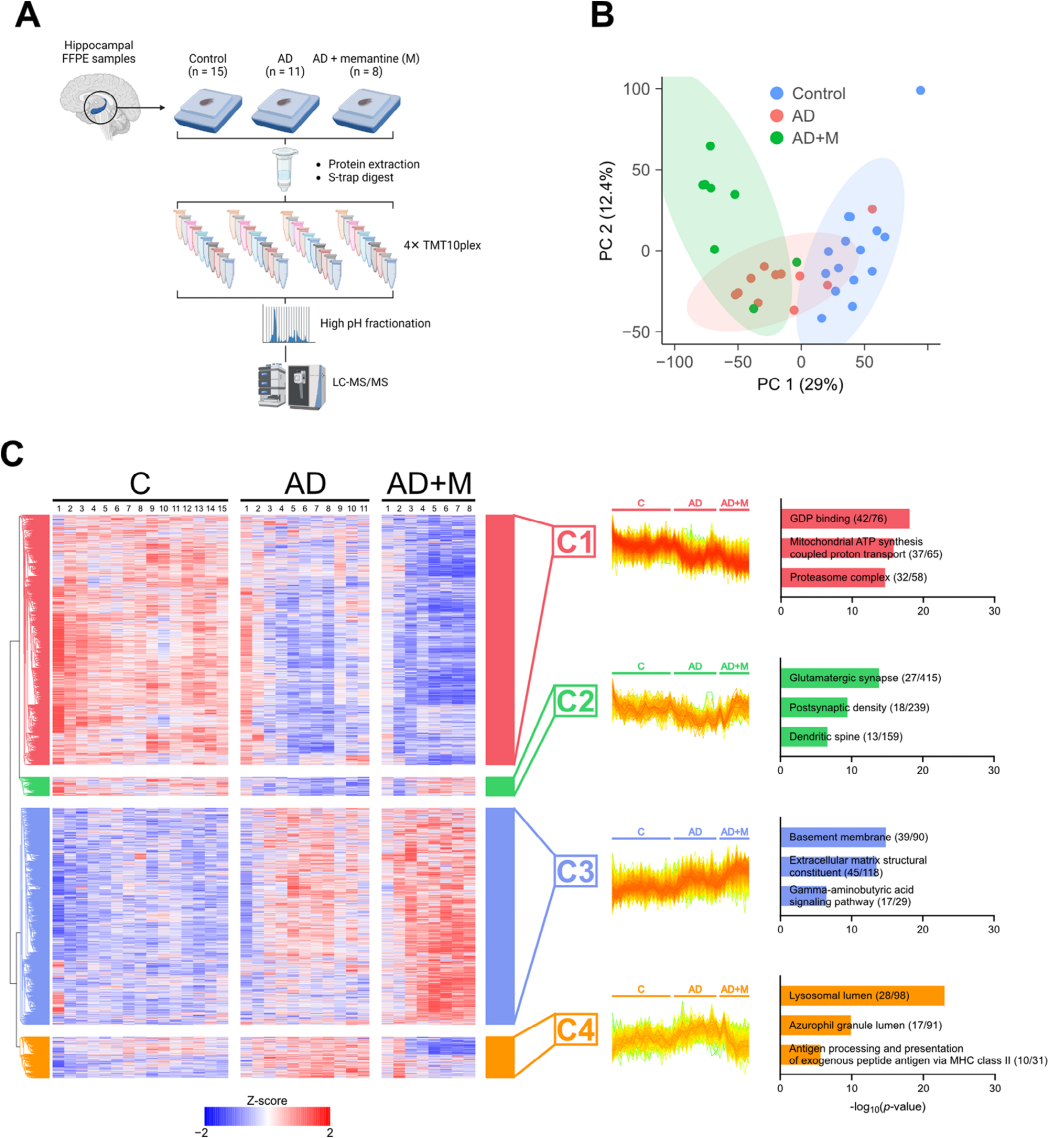
(A) scheme of sample preparation workflow. For details see Supporting information. (B) PCA analysis of patient samples used in the study. (C) Hierarchical clustering of proteins significantly regulated among patient cohorts (ANOVA, permutation‐based FDR <0.05). Clustered proteins were annotated by Gene Ontology (GO) and the relative enrichment of GO term in each cluster was analyzed by DAVID web‐based tool (https://david.ncifcrf.gov/). Graphs on the right show only three terms with lowest FDR and having at least five‐fold change. For each GO term, the number of annotated proteins identified within the respective cluster versus total count of annotated genes in the background database is emphasized.

In total, 9087 proteins were identified, of which 7737 were quantified in all samples (Table S2). To first check the global features of the dataset, samples were compared by principal component analysis (PCA, Figure 1B) and hierarchical clustering (Figure S1). Both analyses confirmed the dominant role of the disease for clustering of the data (AD/AD + M[memantine] vs. controls) with only a minor role of memantine treatment, suggesting that memantine intervention does not have profound effects on the hippocampal proteome of AD patients (Figures 1A and S1). Interestingly, the proteomic profile of one AD patient resembled rather those of healthy controls (labelled as AD 1, Figure S1). Although the patient was diagnosed with AD, re‐examination of hippocampal FFPE tissue by a pathologist was inconclusive. Nevertheless, the sample was kept in the dataset for further interpretation to follow the original clinical diagnosis. To gain deeper insight into the impact of memantine treatment, proteins quantified in all samples were tested by ANOVA (FDR < 0.05) and those found significant were clustered based on their expression profiles in the patient groups (Figure 1C). Memantine treatment had either no effect or slightly amplified the expression trends set by AD in the majority of regulated hippocampal proteins (clusters 1 and 3, C1 and C3, Figure 1C), as was expected based on global inspection of data (Figures 1B and S1), thus further strengthening the finding that memantine does not induce dramatic changes in the hippocampus of AD patients. Notably, however, a relatively small group of proteins responded to memantine intervention in AD patients by increasing (C2) or decreasing (C4) their expression and thus counteracting the effect of AD pathology on their hippocampal levels (Figure 1C).

Clusters defined in Figure 1C were further characterized by Gene Ontology (GO) term enrichment (graphs in Figure 1C). Both mitochondrial and proteasomal proteins were downregulated in the course of AD. The drop in expression remained relatively insensitive to memantine medication (C1, Figure 1C) despite several studies’ reporting memantine‐mediated altering of mitochondrial functions [20, 21, 22, 23, 24]. In the presented data, many proteins involved in oxidative mitochondrial metabolism such as TCA cycle enzymes or components of respiratory complex I (RCI, Figure 2A), RCIII, RCIV, and ATP synthase (Figure S2A) were consistently downregulated in hippocampi of AD patients independently of memantine treatment. The notable exceptions were products of mtDNA‐encoded genes such as subunits of RCI (*MT‐ND1‐6*, Figure 2A), *MT‐CYB*, and *MT‐CO1* (Figure S2A). Interestingly, the subunit .ND2 has been shown to have extra‐mitochondrial function in mediating interaction between Src and NMDA receptors [25, 26] and it is plausible that memantine‐driven perturbation of glutamate signaling in the brain (see below) might affect mitochondrial ND2 expression. The role of upregulation of other mitochondrially‐encoded proteins, however, remains elusive, and it is unclear whether these also participate in glutamate receptor signaling or whether their increased levels come as a by‐product of long mitochondrial polycistronic transcripts [27]. Collectively, such memantine‐dependent upregulation of mitochondrial expression, however, does not seem to improve global metabolic decline (Figures 2A and S2A,B) associated with AD pathology [28, 29].

**FIGURE 2:**
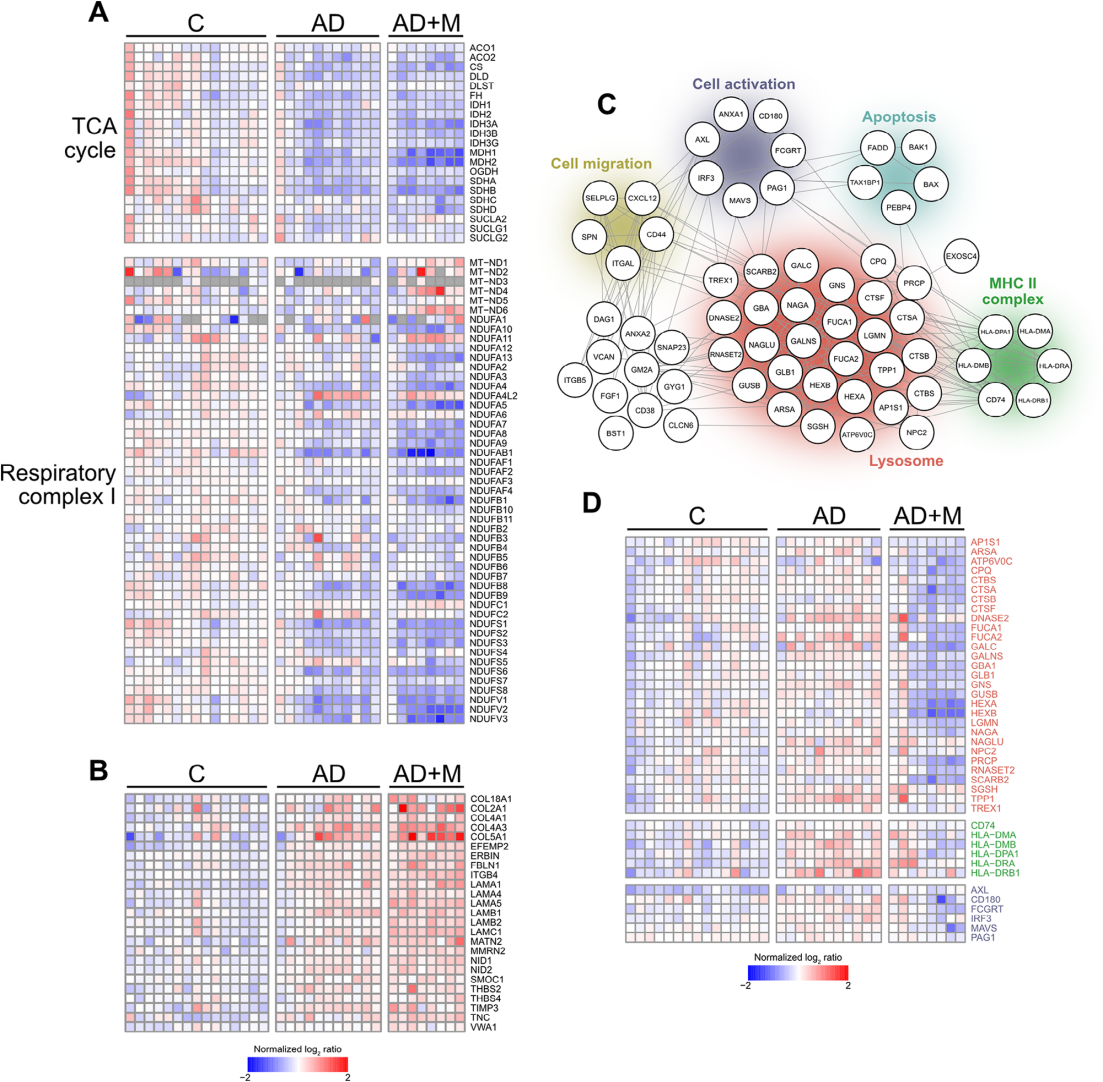
Heatmaps showing expression of (A) proteins involved in TCA and respiratory complex I subunits and (B) basement membrane proteins from C3 cluster in Figure 1C. (C) STRING network (v12.0, score 0.400) containing proteins related to lysosomes and immune system processes allocated in C4 cluster in Figure 1C. (D) Heatmap showing expression of selected lysosomal proteins, components of MHC II system, and proteins related to immune cell signaling with preserved color coding based on (C).

Thickening of the vascular basement membrane (BM) by deposited Aβ is another hallmark of AD [30]. Correspondingly, proteins involved in the formation of BM were upregulated in AD patients (C3 in Figure 1C). Indeed, BM proteins such as collagens, laminins, nidogens, or thrombospondins showed increased expression independently of memantine treatment (Figure 2B), indicating that memantine has no discernible effect on BM pathology during AD.

A small fraction of proteins was upregulated in AD but their levels normalized to those in healthy subjects upon administration of memantine (C4, Figure 1C). Although GO enrichment indicated these are predominantly associated with lysosomes or antigen presentation via MHC II (Figure 1C), closer inspection of the cluster revealed also proteins involved in immune cell signaling, migration, and apoptosis (Figure 2C), suggesting immune cell origin. The major population of immune cells in the steady state brain are microglia. One of their main functions is to maintain brain tissue homeostasis by eliminating tissue debris, for which they possess unique phagosomal machinery [31]. Chronic stimulation of microglia by Aβ plaques in AD, however, leads to their excessive activation, production of proinflammatory cytokines, and increased recruitment of other monocyte‐derived phagocytes, which eventually leads to brain tissue damage contributing to AD pathology [32, 33, 34]. Correspondingly, increased presence of lysosomal enzymes and components of MHC II (HLA) indicated the accumulation of activated phagocytes in the AD hippocampus (Figure 2D). Memantine administration in AD patients not only reverted this expression pattern but led to downregulation of other proteins related to phagocyte activation such as MAVS or IRF3 involved in type I IFN signaling (Figures 2D and S3). Although the effect of memantine on microglia is probably indirect [34] in contrast to lymphocytes [35, 36], these results indicate that memantine can alleviate phagocyte‐mediated inflammation in the AD brain.

Several GO terms linked to proteins upregulated in response to memantine administration were related to synaptic functions (C2 and C3, Figure 1C), consistent with the localization of memantine's primary targets. To investigate these memantine‐dependent synaptic events in more detail, synaptic proteins were identified among the significantly regulated proteins (Figure 1C) using SynGO annotation [37] and subsequently re‐clustered using the same approach (Figure 3A). Overall, memantine administration did not improve the decline of presynaptic proteins observed in AD patients (cluster C2, Figure 3A), as exemplified by proteins involved in synaptic vesicle processing in presynaptic neurons (Figure S4A). Interestingly, however, memantine treatment specifically enhanced the levels of hippocampal postsynaptic components (cluster C1, Figure 3A), including proteins participating in the regulation of postsynaptic receptors, postsynaptic signaling (Figures 3B and S4B), and, notably, neurotransmitter‐sensing receptors of glutamatergic and GABAergic pathways (Figures 3B,C and S5). Indeed, several subunits of iGluRs including the NMDA (GRIN1 and GRIN2B), kainate (GRIK2 and GRIK5, Figure 3C), and AMPA (GRIA2 and GRIA3, Figure S5) receptor family and subunits of mGluRs (GRM1 and GRM2, Figure S5) were upregulated in memantine‐treated AD patients when compared to the unmedicated group. In parallel, enhanced GluR levels were mirrored by a memantine‐mediated increase in expression of glutamate transporters (SLC1A3, SLC17A6 and SLC17A7, Figure S5). The stimulatory effect of memantine on glutamatergic receptor expression was further corroborated in the 5 × FAD transgenic mouse model of AD, where long‐term administration of the memantine also increased the expression of hippocampal GRIN1 and GRIN2B (Figure 3D, compare with Figure 3C), without affecting presynaptic CAMK2A (Figure 3D, compare with Figure S4A).

**FIGURE 3:**
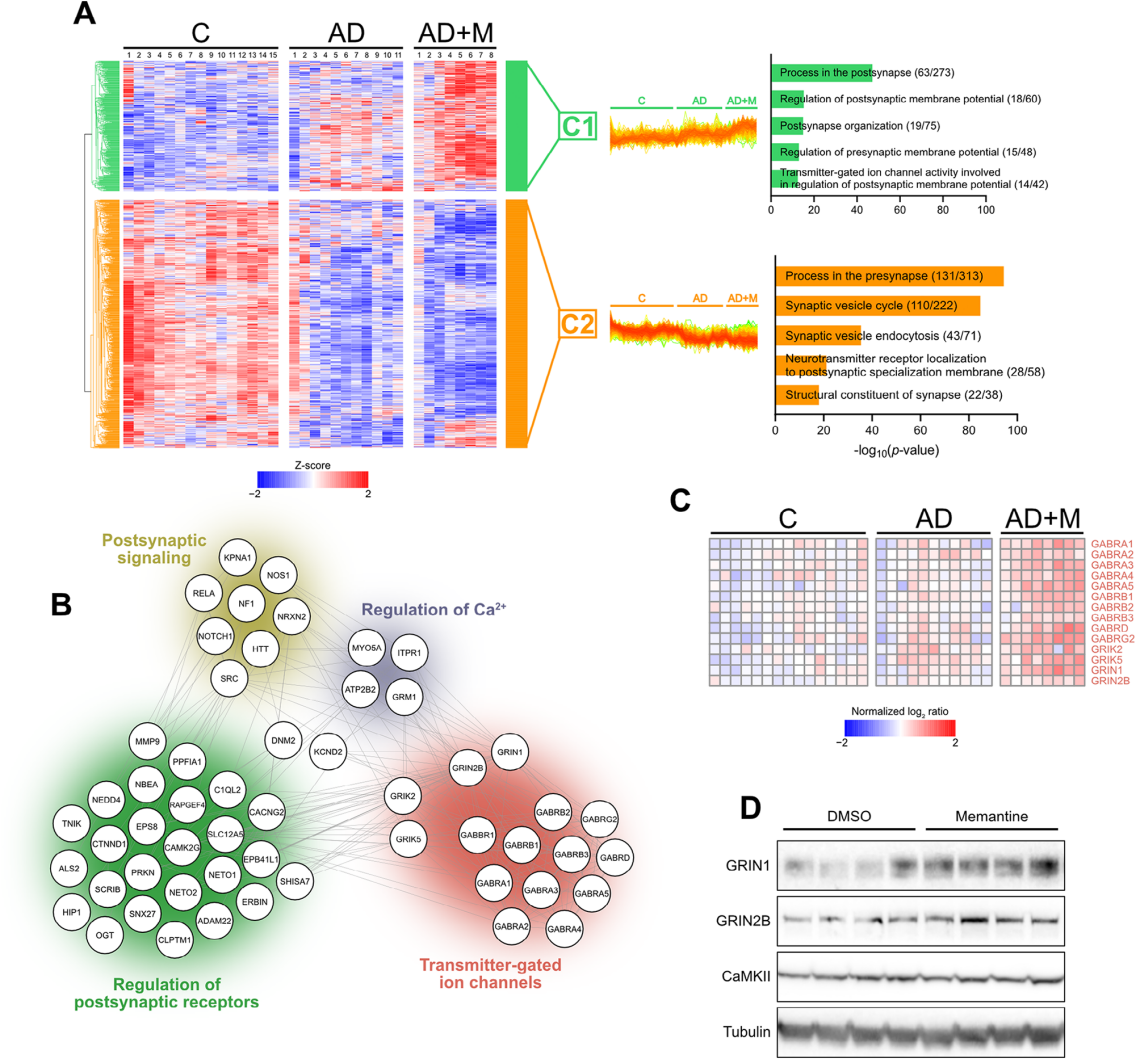
(A) Hierarchical clustering of proteins significantly regulated among patient cohorts (ANOVA, permutation‐based FDR < 0.05) and annotated by SynGO database. Relative enrichment of SynGO terms in each cluster was SynGO web‐based tool (https://www.syngoportal.org/). Graphs on the right show only five terms with lowest FDR and having at least 12‐fold change. For each SynGO term, the number of annotated proteins identified within the respective cluster versus total count of annotated genes in the background database is emphasized. (B) STRING network (v12.0, score 0.400) containing proteins related to processes in the post‐synpase allocated in C1 cluster in Figure 3A. (C) Heatmap showing expression of transmitter‐gated ion channels with preserved color coding based on (B). (D) Western blot analysis of GRIN1, GRIN2B, and CaMKII expression in hippocampi of 5 × FAD transgenic mice treated (or not) with memantine. Tubulin was used as a loading control.

It has been previously shown that the chronic administration of memantine may lead to upregulation of NR2B (GRIN2B) NMDA receptor subunit in rats [38] probably to maintain homeostatic synaptic plasticity via a negative feedback loop [39]. However, the herein observed simultaneous upregulation of other glutamate receptors not directly targeted by memantine indicates that synaptic adaptation to NMDAR blockade involves a more complex reorganization of the glutamatergic pathway, including also activation of antagonistic GABAergic signaling. Indeed, hippocampal expression of ionotropic GABA_A_ (GABRA1‐5, GABRB1‐3, GABRD, and GABRG2; Figure 3C) and metabotropic GABA_B_ (GABBR1‐2; Figure S5) receptor subunits, along with proteins involved in GABA metabolism (GAD1 and SLC32A1; Figure S5), was also elevated in AD patients following memantine administration, thus mirroring the expression patterns of glutamatergic receptors. Considering the fact that memantine has no affinity for GABA receptors, such upregulation of GABA_A_ and GABA_B_ receptors could be a consequence of neural excitatory/inhibitory homeostasis ensuring a balance in excitatory (glutamate) and inhibitory (GABA) pathways [40, 41].

In summary, the presented data show that memantine treatment has a subtle yet distinct impact on the hippocampal proteome in AD patients (Figures 1B,C and S1). Although the majority of AD‐regulated proteins remained relatively unaffected (Figures 2A,B and S2A,B), memantine medication selectively mitigated the proteomic pattern of activated phagocytes (Figures 2C,D and S3) and enhanced the expression of synaptic components involved in glutamate and GABA neurotransmission (Figures 3A–D, S4, and S5). There are, however, several limitations that must be considered when interpreting these findings. First, the patient cohorts are relatively small and variable. Only little is known about the patients' other medications, severity of AD in the time of autopsy, and the time of autopsy itself which may affect the proteome. Although memantine‐dependent expression of NMDAR subunits was validated using an AD murine model in vivo, the expression profiles of individual proteins needs to be confirmed either by more targeted approaches or in a larger cohort of patients. In addition, it is unknown whether the observed upregulation of synaptic receptors correlates with their enhanced activity or whether it reflects accumulation of their inactive forms. Finally, the current data describe the proteomic landscape at a single time point without any information about the dynamics of protein expression. This prevents any conclusions with regard to causality of observed effects, for example, whether blockade of NMDAR signaling directly stimulates the expression of GABAergic receptors or whether there are other intermediate events. Nevertheless, the herein reported findings strongly indicate that memantine treatment in AD patients not only alleviates excitotoxic stress by inhibiting NMDA receptor activity, but also triggers broader adaptations in synaptic signaling that reflect modulation of plasticity at the network level. Collectively, the presented data and accompanying dataset describe new details and provide novel insights about the complex mode of action of memantine in the hippocampus of AD patients.

## Author Contributions

**I.F**. conceptualization, data curation, methodology, investigation, visualization, writing – original draft. R**.K**. data curation, validation, methodology, writing – review & editing. D**.F., M.C., K.Ho., K.Ha,** validation, methodology, M**.H**. conceptualization, resources, writing – review & editing, J**.S., M.M., R.R., R.M., A.R**. Resources, Writing—Review & Editing**, O.S**. Conceptualization, Writing—Review & Editing, Funding acquisition, Project administration

## Ethics Statement

The part of study involving humans has been approved by the Ethical Committee of the University Hospital Hradec Kralove (ref. 201906S15P) and the part involving animals was approved by the Animal Care and Use Committee of the National Institute of Mental Health (MZDR 19618/2021‐5/OVZ). Due to the fact that autoptic material is used in this retrospective fully anonymized study, informed consent is not required. The manuscript does not contain any individual person's data.

## Conflicts of Interest

The authors declare that they have no competing interests

## Supporting information



Supporting information

Supporting information

## Data Availability

The mass spectrometry proteomics data have been deposited to the ProteomeXchange Consortium (http://proteomecentral.proteomexchange.org) via the PRIDE partner [19] repository with the dataset identifier PXD052985. Reviewer access details: Log in to the PRIDE website using the following details: Project accession: PXD052958 Token: GycP5undMMwr
