# Supplementary material for: Memantine Administration Enhances Glutamatergic and GABAergic Pathways in the Human Hippocampus of Alzheimer's Disease Patients
Source: Proteomics. 2025 Jul 9;25(15):42–9. doi: 10.1002/pmic.70006 (PMC12329391; doi:10.1002/pmic.70006)
Supplement: Supplementary file 1 — Supporting information [file PMIC-25--s001.docx]

**Supplementary file**

**Title:** Memantine administration enhances glutamatergic and GABAergic pathways in human hippocampus of Alzheimer´s disease patients.

Ivo Fabrik^1^, Rudolf Kupcik^1^, Daniela Fabrikova^1^, Marketa Chvojkova^3,4^, Kristina Holubova^3,4^, Kristina Hakenova^3,4^, Martin Horak^5^, Jiri Soukup^2,8,9^, Monika Manethova^2^, Robert Rusina^6^, Radoslav Matej^7^, Ales Ryska^2^, Ondrej Soukup^1^*

^1^ Biomedical Research Centre, ^2^ The Fingerland department of Pathology, University Hospital Hradec Kralove, Sokolska 581, 500 05 Hradec Kralove, Czech Republic

^3^ National Institute of Mental Health, Topolová 748, 250 67 Klecany, Czech Republic and ^4^Third Faculty of Medicine, Charles University Ruska 87, 100 00 Prague 10, Czech Republic

^5^ Institute of Experimental Medicine of the Czech Academy of Sciences, Videnska 1083, 14220 Prague 4, Czech Republic

^6^ Clinic of Neurology, ^7^ Department of Pathology and Molecular Medicine 3^rd^ Faculty of Medicine Charles University and Thomayer University Hospital, Videnska 800, 14220 Prague 4,

^8^ Department of Pathology, Military University Hospital Prague, U Vojenske Nemocnice 1200, Praha 6, 169 02, Prague, Czech Republic.

^9^ Department of Pathology, Charles University, First Faculty of Medicine and General University Hospital in Prague, Studnickova, 2039, 128 00, Nove Mesto, Prague, Czech Republic

*** Address correspondence and reprint requests to:

Ondrej Soukup, Biomedical Research Centre, University Hospital Hradec Kralove, Sokolska 581, 500 05 Hradec Kralove, Czech Republic, Email: ondrej.soukup@fnhk.cz

**Methods**

*Animals*

Male 5xFAD mice (B6.Cg-Tg(APPSwFILon,PSEN1M146LL286V)6799Vas/Mmjax; Jackson Laboratory, USA), which carry five familial Alzheimer's disease (FAD) mutations resulting in the overexpression of human amyloid-β precursor protein and enhanced γ-secretase activity, were used. The mice were 4 months old at the beginning of the experiment (24–34 g). They were housed in pairs in transparent plastic individually ventilated cages (32 × 17 × 12 cm) in an animal room of the National Institute of Mental Health, Klecany, Czech Republic, with constant temperature (22 ◦C), humidity (40–70%), and a 12 h light/dark cycle. Water and food were available ad libitum. The experiments were conducted in accordance with the guidelines of European Union directive 2010/63/EU and Act No. 246/1992 Coll. on the protection of animals against cruelty and were approved by the Animal Care and Use Committee of the National Institute of Mental Health (MZDR 19618/2021-5/OVZ).

Memantine (memantine hydrochloride, catalog number PHR1886, Sigma-Aldrich, St. Louis, MO, USA) was dissolved in the vehicle and administered intraperitoneally at the dose of 5 mg/kg/day (injection volume 10 ml/kg/day). The vehicle contained 1% dimethyl sulfoxide (Carl Roth, Karlsruhe, Germany) in physiological saline. Control animals received corresponding volume of the vehicle.

The mice were pseudo-randomly assigned to two treatment groups: memantine and vehicle. Treatment was initiated at 4 months of age. The drugs were administered daily in three 28-day cycles. Specifically, the drugs were administered for 28 days, followed by a 14-day drug-free period for evaluation of behavioral effects. Treatment was then resumed for another 28 days, followed by an additional 14-day drug-free interval. Subsequently, the drugs were administered for a final 28-day period, followed by a 10-day drug-free phase, after which the mice were sacrificed. The mice were over 8 months old at the time of the sacrifice. In particular, the mice were anesthetized in isoflurane inhalation anesthesia (Forane, AbbVie, Czech Republic), decapitated, brains were rapidly removed from the skull and rinsed in physiological saline. The left hippocampus was dissected, weighed, frozen on dry ice, and stored at −80 ◦C before sample preparation and analysis.

*Western blot*

Hippocampal tissues from 4 mice treated with DMSO (controls) and 4 memantine-treated mice were resuspended in 3% sodium deoxycholate/100 mM TEAB (5 µl per mg of tissue) and homogenized using ultrasonic transducer (Hielscher). Homogenates were centrifuged (14000g, 15 min, 10 °C) and protein content in supernatants was determined by BCA assay (Sigma). Denatured and reduced samples were separated on NuPAGE 4-12% Bis-Tris Gels (Thermo) and transferred to 0.45μm PVDF membranes. Blots were blocked by milk and incubated with primary antibody overnight followed by secondary antibody conjugated with horseradish peroxidase (HRP; Thermo). Bands were visualized by ECL (Amersham) and captured by Azure c280 (Azure Biosystems). The following primary antibodies were used: anti-tubulin (cat# ab6046, Abcam), anti-GluN1 (cat# 5704, Cell Signaling), anti-GluN2B (cat# 4212, Cell Signaling), and anti-CaMKII-α (cat# 11945, Cell Signaling).

*Patients*

The FFPE samples were provided from two biobanks. The Fingerland department of Pathology and The Institute of Pathology and Molecular Medicine 3rd Faculty of Medicine Charles University and Thomayer University Hospital provided the standard FFPE control samples (15) with average age 64.9±6.3, the memantine untreated AD samples (11) with average age 81.8 ± 11.3 and memantine treated AD (AD+M) samples (8, 78.5 ±5.2). The tissue used was right human hippocampus (rostral part)

*FFPE tissue homogenization*

For whole FFPE tissue blocks, samples were cut into small pieces and transferred entirely into 2 ml tubes. FFPE tissue sections (samples AD+M 3-8) were directly transferred into tubes. All samples were overlayed by 1 ml of xylene, vortexed, and incubated in thermomixer at RT for 5 min. Tubes were briefly centrifuged, supernatant was discarded, and xylene wash was repeated three times. FFPE slices were then overlayed by 1 ml of ethanol (EtOH) and the wash procedure was repeated as described above. Following final wash by EtOH, tissue slices were dried in Speed-vac and resuspended in Protein Extraction Buffer (FASP Protein Digestion Kit, Abcam) containing TCEP at a ratio of 1 ml of buffer per 50 mg of dried tissue. Tissue samples were then homogenized in disperser (IKA) and incubated at 105 °C for 30 min. After cooling to RT, samples were centrifuged (15000 g/10 min) and supernatants were collected for further processing.

*Protein digest and TMT labeling*

Protein concentrations in homogenates were measured by BCA assay (Sigma Aldrich, USA) and 20-100 µg from each sample was taken for digest by S-trap. Briefly, samples were diluted by S-trap SDS lysis buffer (final concentration 5% SDS/50 mM TEAB) and alkylated by 30 mM iodoacetamide for 20 min in dark. Samples were then acidified by phosphoric acid (final concentration 2.5%), mixed with 6 volumes of 90% MeOH/100 mM TEAB (pH 7.55), and loaded onto S-trap micro columns (Protifi) by centrifugation (4000 g/30 s). Columns were washed four times by 150 µl of 90% MeOH/100 mM TEAB (pH 7.55) and trapped proteins were digested by 2 µg of Lys-C/trypsin mixture (Promega) in 50 mM TEAB at 37 °C overnight. Peptides were eluted by consecutive 50 µl washes of 50 mM TEAB, 0.2% formic acid (FA), and 50% ACN (4000g/1 min) applied to S-trap columns. Eluates were combined, frozen at -80 °C, and lyophilized for 2 days. Lyophilized peptides were dissolved in 55 µl of 100 mM TEAB and their concentration was determined by Pierce Quantitative Fluorometric Peptide Assay (Thermo). Samples were distributed into 4 multiplexes (each containing also pooled reference sample, see Table S2) and peptides from each sample were labeled for 1h by the respective TMT10plex tag (Thermo, Lot: YC367338) dissolved in ACN before the reaction was quenched by the addition of hydroxylamine. Then the samples from the respective multiplexes were combined, desalted using Pierce Peptide Desalting Spin Columns (Thermo), and dried in Speed-vac.

*High pH HPLC fractionation*

Samples were re-dissolved in mobile phase A (2% ACN/10mM NH_4_FA) and 84 µg of labeled peptides were injected on UltiMate 3000 RSLC system (Thermo). Peptides were separated using XBridge BEH column C18, 2.5 µm, 2.1 µm x 150 mm (Waters) in linear gradient of mobile phase B (80% ACN/10 mM NH_4_FA) at flow rate of 0.3 mL/min. Gradient was running from 0% B to 2% B in 2 min, followed by 2% B to 20% B in 9 min, from 20% B to 50% B in 41 min and from 50% B to 52% B in 5.5 min. In total, gradient time was 57.5 min. Fractions were collected in 96-well polypropylene plate (Agilent Technologies) at 45 s intervals from 3.7 to 57.7 min using FC 204 fraction collector (Gilson) yielding 72 fractions. Collected fractions were subsequently concatenated into 24 fractions, dried in vacuum concentrator and stored at -80 °C until nLC-MS/MS analysis.

*nLC-MS/MS analysis*

Each fraction was re-dissolved in loading solvent (2% ACN/0.1% TFA) and 0.8 µg of peptide material was injected in UltiMate 3000 RSLCnano system (Thermo) in duplicate. The analytical system was equipped with PepMap100 C18, 3 µm, 100 Å, 75 µm × 20 mm trap column and PepMap RSLC C18, 2 µm, 100 Å, 75 µm × 250 mm analytical column (both Thermo). All fractions were loaded in trap column at flow rate of 5 µL/min for 5 min. Peptides were separated in linear gradient of mobile phase A (2% ACN/0.1% FA) and B (80% ACN/0.1% FA) running from 2% B to 34.5% B in 70 min followed by 34.5% B to 45% B in 10 min at a flow rate of 250 nL/min. The eluted peptides were introduced via Nanospray Flex ion source into Orbitrap Exploris 480 mass spectrometer which was equipped with FAIMS Pro Duo interface (Thermo). The ionization voltage was set at 1,8 kV and capillary temperature was maintained at 280 ^°^C. The Orbitrap Exploris 480 was operated in data-dependent acquisition (DDA) mode with the following settings: full-scan mass spectra were collected with m/z range 300-1400, resolution of 60,000 (at m/z 200), AGC target value of 300%, and maximum IT of 25 ms. After MS1 scan, the 10 most intense precursor ions were selected for MS/MS fragmentation (isolation width 1.3 m/z, precursor fit 70%) and fragmented (NCE 35%) by HCD. MS2 analysis was performed with resolution of 15,000 (at m/z 200), 34 ms maximum IT, enabled TurboTMT, and with AGC target 200%. Charge states < 2 and > 5 were excluded and dynamic exclusion was set to a duration of 17 s after fragmentation n=1 times. FAIMS Pro Duo was operated under a gas flow of 4.6 L/min, with compensation voltages (CVs) set at −45 V and −60 V.

*Data interpretation*

Raw files were first converted to mzML format by ProteoWizard MSConvert (v3.0.23345) and then interpreted using MSFragger (v4.0) integrated into FragPipe (v21.1). The settings of the search were based on “TMT10-bridge” workflow with slight modifications. Precursor and fragment mass tolerance was 20 ppm with mass recalibration and parameter optimization enabled. Trypsin was set as a protease with 2 allowed missed cleavages and minimal peptide length of 7 amino acids. Methionine oxidation (+15.9949), N-terminal acetylation (+42.0106), TMT modification of N-terminal and serine (+229.16293) and clipping of N-terminal methionine were set as variable modifications. Carbamidomethylation of cysteine (+57.02146) and TMT modification of lysine (+229.16293) were set as fixed modifications. MS/MS spectra were searched against Human Reference proteome database downloaded from Uniprot (February 16, 2024; 82499 sequences) combined with FragPipe-supplied contaminants and decoy sequences. MSBooster (v 1.1.28) was used for correlation of predicted RT and MS/MS spectra followed by rescoring and validation of PSMs by Percolator (v 3.06.4) ProteinProphet and Philosopher (v5.1.0) were used for protein inference and filtering of proteins at 1% FDR. Protein quantification was carried out by TMT-Integrator (v5.0.7) taking razor and unique peptides with minimal PSM probability of 0.9. Data were normalized by median centering on protein level using pooled reference sample labeled by TMT10-131 tag in each multiplex (Table S2). To define protein clusters based on the expression profile (Figure 1C), log2-ratios of proteins quantified in all samples were first subjected to ANOVA (permutation-based FDR <0.05) in Perseus (v1.6.2.1). Significant hits were then Z-transformed and clustered by hierarchical clustering using ‘pheatmap’ package in R. Protein members from each cluster were then used for analysis of Gene Ontology term enrichment in DAVID web-based tool (<https://david.ncifcrf.gov/>). For each cluster, only three GO terms with lowest FDR and having at least 5-fold change enrichment and at least 5 identified members were reported (Figure 1C). To define synaptic protein clusters (Figure 3A), significant hits from ANOVA (see above) were first filtered for those annotated by SynGO database (<https://www.syngoportal.org/>) before clustering as described above. Synaptic proteins from each cluster were then used for analysis of SynGO term enrichment in SynGO web-based tool. For each cluster, only five SynGO terms with lowest FDR and having at least 12-fold change enrichment and at least 5 identified members were reported (Figure 1C).

Supplementary Table S1: Cohort description

| Patient | Sex | Age | Memantine treatment | Used FFPE material | Weight of deparaffinized dried sample (mg) |
| --- | --- | --- | --- | --- | --- |
| C 1 | M | 74 | N/A | Whole FFPE block | 39.5 |
| C 2 | M | 60 | N/A | Whole FFPE block | 148.1 |
| C 3 | M | 62 | N/A | Whole FFPE block | 80.5 |
| C 4 | M | 70 | N/A | Whole FFPE block | 109.6 |
| C 5 | M | 68 | N/A | Whole FFPE block | 61.9 |
| C 6 | F | 57 | N/A | Whole FFPE block | 49.5 |
| C 7 | M | 72 | N/A | Whole FFPE block | 140.7 |
| C 8 | M | 59 | N/A | Whole FFPE block | 178.6 |
| C 9 | M | 71 | N/A | Whole FFPE block | 173.8 |
| C 10 | M | 72 | N/A | Whole FFPE block | 168.2 |
| C 11 | F | 63 | N/A | Whole FFPE block | 86.4 |
| C 12 | M | 63 | N/A | Whole FFPE block | 133.5 |
| C 13 | F | 53 | N/A | Whole FFPE block | 88.0 |
| C 14 | M | 66 | N/A | Whole FFPE block | 114.4 |
| C 15 | F | 67 | N/A | Whole FFPE block | 62.1 |
| AD 1 | F | 74 | N/A | Whole FFPE block | 103.8 |
| AD 2 | M | 83 | N/A | Whole FFPE block | 83.7 |
| AD 3 | M | 74 | N/A | Whole FFPE block | 90.1 |
| AD 4 | F | 59 | N/A | Whole FFPE block | 42.7 |
| AD 5 | F | 85 | N/A | Whole FFPE block | 317.1 |
| AD 6 | F | 86 | N/A | Whole FFPE block | 203.5 |
| AD 7 | M | 88 | N/A | Whole FFPE block | 217.3 |
| AD 8 | M | 87 | N/A | Whole FFPE block | 112.2 |
| AD 9 | M | 87 | N/A | Whole FFPE block | 132.3 |
| AD 10 | F | 103 | N/A | Whole FFPE block | 147.2 |
| AD 11 | M | 74 | N/A | Whole FFPE block | 64.0 |
| AD+M 1 | F | 81 | 3 years | Whole FFPE block | 48.7 |
| AD+M 2 | M | 84 | Unknown* | Whole FFPE block | 163.7 |
| AD+M 3 | M | 87 | 7 years | Tissue sections (slices) | 5.4 |
| AD+M 4 | F | 74 | 17 months | Tissue sections (slices) | 3.8 |
| AD+M 5 | M | 72 | 6 years | Tissue sections (slices) | 8.0 |
| AD+M 6 | M | 77 | 3.5 years | Tissue sections (slices) | 5.3 |
| AD+M 7 | M | 77 | 13 months | Tissue sections (slices) | 4.4 |
| AD+M 8 | M | 72 | 15 months | Tissue sections (slices) | 5.0 |

*Administration of memantine was confirmed in the clinical history without further details

| 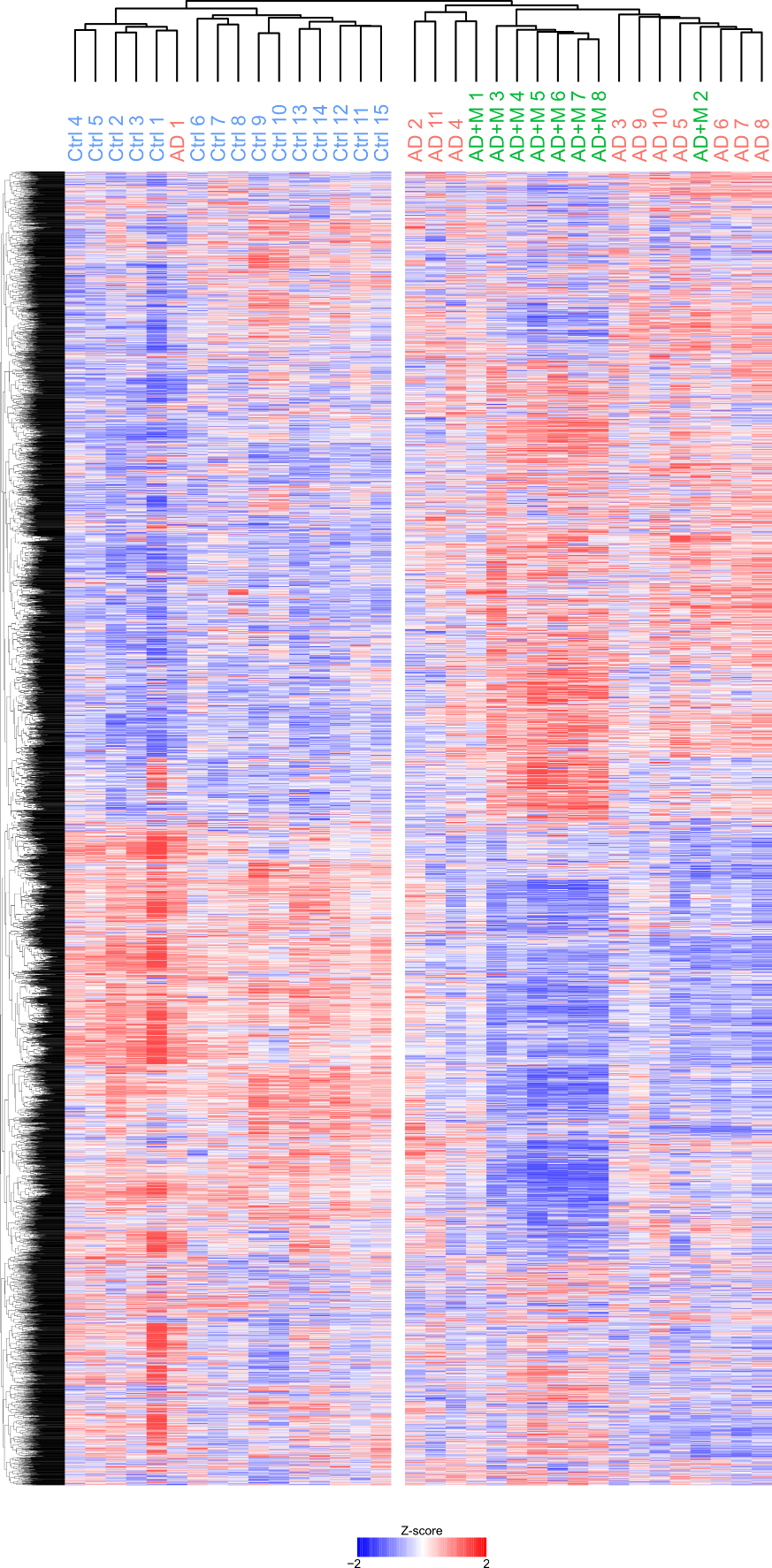 |
| --- |
| **Supplementary Figure S1:** Hierarchical clustering of patients based on proteins quantified in all samples. The color of the sample corresponds to Figure 1B. |

| 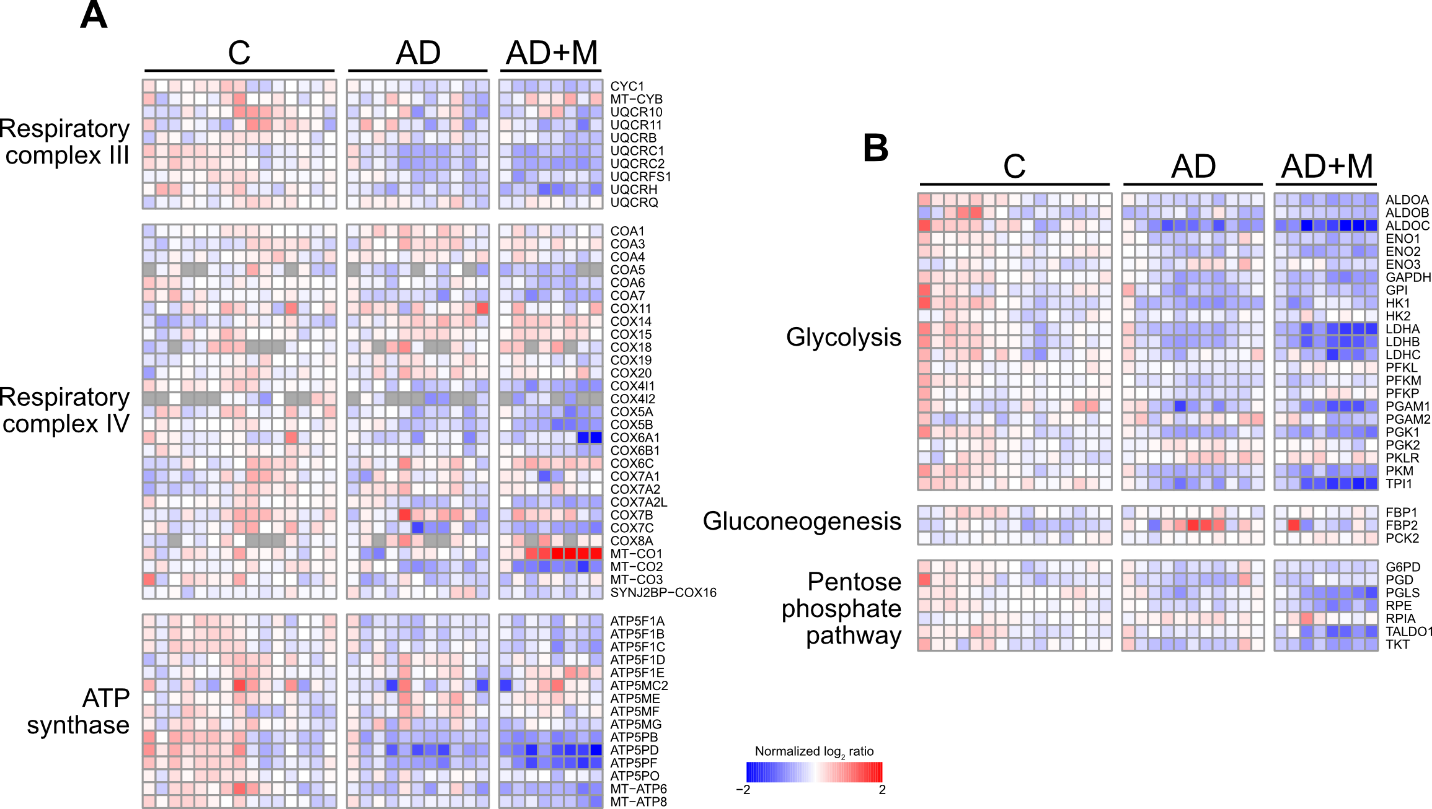 |
| --- |
| **Supplementary Figure S2:** Heatmaps showing the expression of (A) respiratory complex III, IV and ATP synthase subunits and (B) protein involved in glycolysis, gluconeogenesis, and pentose phosphate pathway. |

| *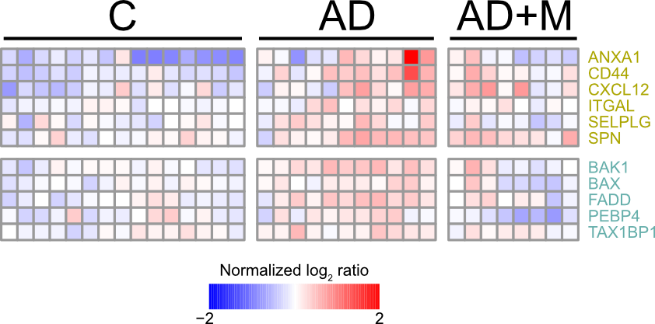* |
| --- |
| **Supplementary Figure S3:** Heatmap showing the expression of selected proteins involved in immune cell migration and apoptosis with preserved color coding based on Figure 2C. |

| *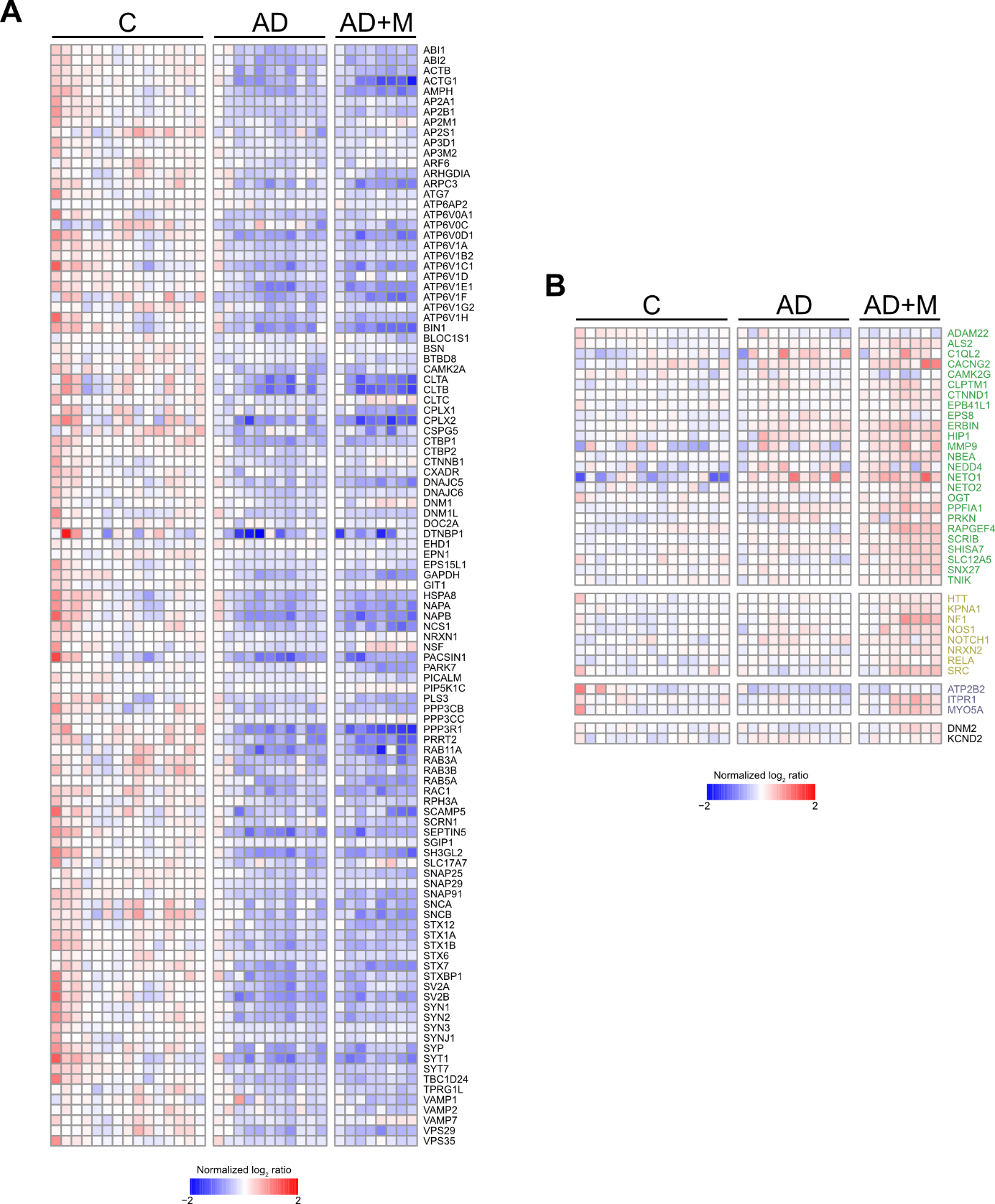* |
| --- |
| **Supplementary Figure S4:** Heatmap showing the expression of (A) proteins related to synaptic vesicle cycle allocated in C2 cluster in Figure 3A and (B) proteins related to regulation of postsynaptic receptors, postsynaptic signaling, and intracellular Ca^2+^ levels in postsynaptic neurons with preserved color coding based on Figure 3B. |

| *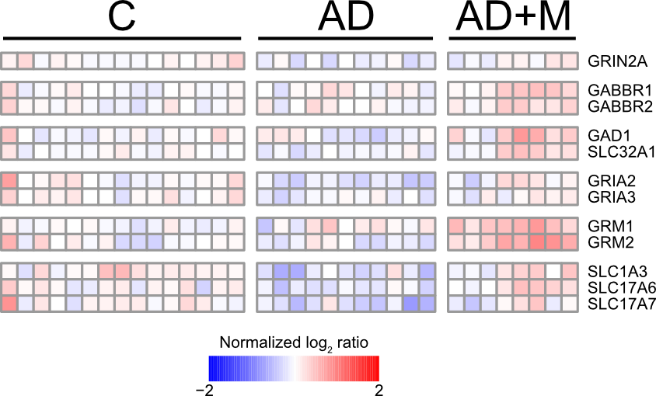* |
| --- |
| **Supplementary Figure S5:** Heatmap showing the expression of other ionotropic and metabotropic glutamate and GABA receptors, transporters, and GABA-synthesizing enzyme GAD1. |
